# Quantum‐Dot‐Mediated Light Field Imaging by Photocurrent Scanning Force Microscopy

**DOI:** 10.1002/nap2.70057

**Published:** 2026-03-24

**Authors:** Florian Küstner, Andreas Hohenau, Harald Ditlbacher, Joachim R. Krenn

**Affiliations:** ^1^ Institute of Physics University of Graz Graz Austria

**Keywords:** photocurrent, photocurrent atomic force microscopy, plasmon, quantum dots

## Abstract

Light field distributions on sample surfaces are mapped by detecting the photocurrents locally induced in a monolayer of colloidal perovskite‐capped PbS quantum dots with a conductive scanning force microsope. The signal levels depend on the illuminating light irradiance according to a power law and the spatial resolution is found to be bias‐dependent in the range of 30–100 nm. We image, first, the Airy intensity pattern of a tightly focused laser beam and, second, the light field distribution in a plasmonic nanoparticle array. With isotropic light absorption and the option of tailoring quantum dots for specific charge generation and transport properties, quantum dots can provide a reliable and generic current read‐out scheme for light field imaging, detection, and sensing.

## Graphical abstract

A colloidal quantum dot layer converts light field patterns to local photocurrents that are probed by conductive atomic force microscopy. A spatial resolution of 30–100 nm is demonstrated by imaging a focus Airy pattern and the light field distribution within a plasmonic nanoparticle array.

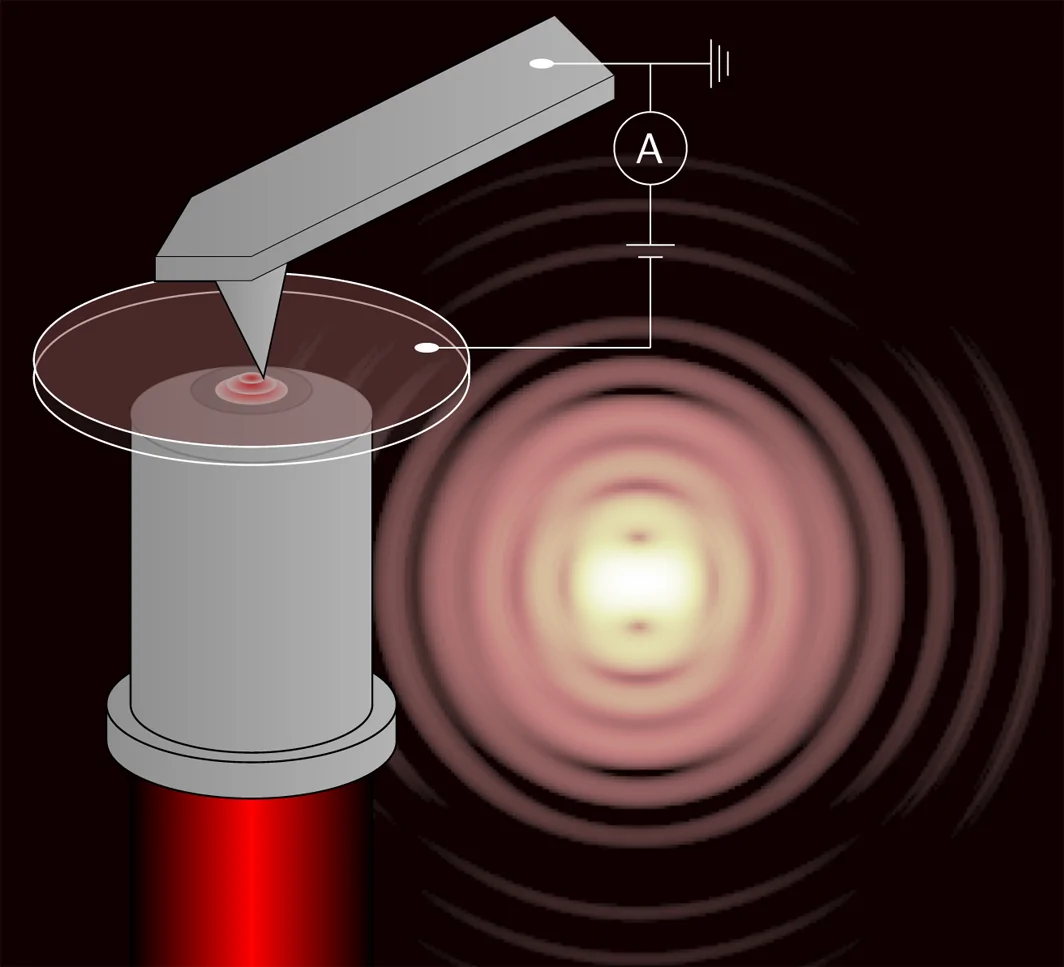

## 1 Introduction

Atomic force microsopy (AFM) with conductive tips can combine topographic imaging with local current detection. Applied to light‐induced currents, photocurrent AFM (pcAFM) probes local charge generation and transport  [1], information sought for, for example, novel photodetecting or photovoltaic materials  [2]. For PbS colloidal quantum dots, pcAFM was recently used to quantitatively retrieve local electronic properties as barrier height or diffusion constants with only a few quantum dots contacted in parallel  [3]. These measurements relied on stable photocurrents and excellent electric contacts to PtIr‐coated AFM tips, arguably enabled by exchanging the organic ligands of the dots by an ultrathin perovskite (${\text{MAPbI}}_{3}$) layer  [4]. While detecting the electronic properties on a sample surface with nanoscale resolution is the central purpose of pcAFM, it should not go unnoticed that the photocurrent signals can be used to map light field distributions, given photosensitive surfaces that provide homogeneous charge generation and transport rates. The purpose of our study is to show the feasibility of this approach and provide an analysis of the spatial resolution. Compared to optical methods as scanning near field optical microscopy (SNOM), pcAFM has the advantage of a truly local and isotropically sensitive detection scheme, unhampered by background light giving rise to interference in the SNOM detection path or polarization‐sensitive probes. Clearly, in view of (scattering) SNOM  [5] as a matured technique that includes phase detection  [6] and that has also been extended to the local excitation of photocurrents (photocurrent nanoscopy  [7, 8]), our study can just provide a first step toward complementing existing schemes with pcAFM imaging.

PbS/${MAPbI}_{3}$ quantum dots are well suited for photodetection mapping as they can be deposited as highly homogeneous and compact monolayers and they absorb light isotropically (when of spherical geometry). In addition, they provide an efficient and stable conductive contact to PtIr‐covered pcAFM tips  [3]. Here, we apply monolayers of PbS/${MAPbI}_{3}$ quantum dots to map, first, the Airy intensity pattern of a laser focus and, second, the light field distribution in between regularly arranged plasmonic nanoparticles. We find the photocurrents from the quantum dots to follow a power law dependence on the light irradiance with an exponent of 0.64, which we use for data normalization. We have consistently measured exponents between 0.60 and 0.69 for PbS/perovskite quantum dots before  [3, 9], which falls in the expected range between 0.5 and 1, due to contributions from bimolecular and monomolecular recombination, respectively  [10]. A quantitative analysis of this value by disentangling the respective roles of the dynamics of carrier generation, transport, and recombination as well as carrier traps is however beyond the scope of this study. Further, we find a bias‐dependent spatial resolution, which we estimate to be in the range of 30–100 nm for zero external bias.

## 2 Experimental

The pcAFM equipped with a standard PtIr‐coated cantilevered silicon tip (Bruker SCM‐PIT‐V2) was installed on an inverted optical microscope. The measurements were done in contact mode with a normal force of 1 nN. Photocurrents were measured with an I/V amplifier (1 nA/V, root‐mean‐square noise level 55 fA), with a bias applied to the sample and the tip grounded. The beam of a stabilized helium neon laser (wavelength 633 nm) was expanded to a diameter of 10 mm and focused through a microscope objective with a numerical aperture (N.A.) of 0.4 or 1.45. With the focus position fixed, either the sample or the tip was scanned (Figure 1a).

**FIGURE 1:**
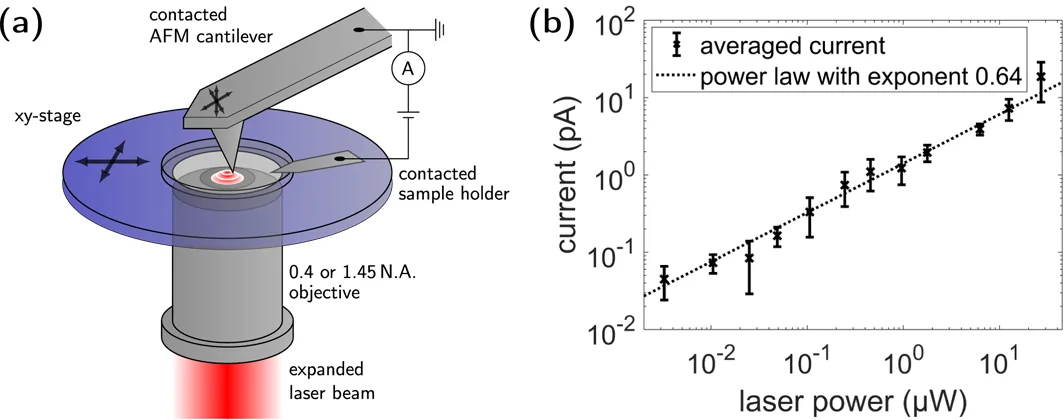
(a) Sketch of the pcAFM setup with a PtIr coated tip. The expanded beam of a 633‐nm wavelength laser is focused through a 0.4 or a 1.45 numerical aperture objective onto the sample. Either the sample stage or the tip is scanned, as indicated by the arrows. The photocurrent between the grounded tip and the sample is measured with an I/V amplifier. (b) Power law dependence of the photocurrent deduced from contact mode sample scans (5 × 5 $\mu m^{2}$) with the PtIr tip in the focus of a 0.4 N.A. objective. The error bars indicate the standard deviation with respect to the line averages of the corresponding pcAFM maps.

A 0.17 mm thick glass slide was used as the substrate, covered with a 20‐nm thick indium tin oxide (ITO) layer deposited by electron beam physical vapor deposition, followed by annealing at 300°C and air plasma cleaning. A compact monolayer of PbS quantum dots with a ${\text{MAPbI}}_{3}$ shell about 1 nm thick (PbS diameter 3.2 nm, synthesized as reported before  [3]) was deposited by spin coating at 2000 rpm a solution of 10 g/L of PbS/${MAPbI}_{3}$ quantum dots in 2,6‐difluoropyridine. Some protruding nanoscale features are observed, presumably due to quantum dot agglomeration, see Supporting Information S1: Figure S1. The gold nanoparticle array sample was prepared by electron beam lithography, exposing a 100‐nm thick poly(methyl methacrylate) layer on glass/ITO, followed by chemical development, gold deposition by resistively heated physical vapor deposition, lift‐off, and the same quantum dot deposition procedure as before. The cuboid‐shaped nanoparticles with a base area of 150 nm by 80 nm and a height of 40 nm are aligned in a 300‐nm pitch square lattice. The numerical simulation of the field distributions within the particle array was done with the MNPBEM toolbox  [11] with the dielectric function of gold from ref. [12].

## 3 Results and Discussion

### 3.1 Photocurrent Dependence on Laser Power

As we use a PtIr probe that is not resonant in the red wavelength range, the reflected light from the tip is just locally reducing the intensity at the sample surface, in proportion to the irradiance. We thus do not expect the tip to have a significant influence on the sample. We first measure the dependence of the photocurrent on the laser power that is expected to follow a power law with an exponent between 0.5 and 1  [13]. For PbS/${MAPbI}_{3}$ quantum dot layers between lateral electrodes, values between 0.60 and 0.69 were reported  [9] and for PbS/${MAPbI}_{3}$ quantum dot monolayers on ITO probed by pcAFM, we recently found an exponent of 0.64  [3]. As depicted in Figure 1b, we retrieve the very same value for our sample (i.e., the quantum dot monolayer on the ITO/glass substrate), for laser powers spanning more than three orders of magnitude. These measurements were done at zero external bias so that the photocurrent is driven only by the built‐in voltage of 0.8±0.1 V (see Supporting Information S1: Section 2), that is, basically the work function difference of ITO and PtIr  [3]. The tip was placed in the center of the focus of the 0.4 N.A. objective, the sample was scanned over an area of 5×5 $\mu m^{2}$, and the photocurrent was averaged over the scan area. With this data, the photocurrents can be linearized for proportional light level detection or comparison with simulations. However, the sublinear photocurrent response might be advantageous when wider measurement ranges than available with a linear response before detector saturation are required.

### 3.2 Photocurrent Mapping of Laser Focus

Figure 2a shows the tip‐scanned pcAFM map of the focus of the 1.45 N.A. objective, acquired with zero external bias and a laser power (before the objective) of 0.4μW. Although the center and part of the lobes of the Airy pattern can be recognized, the current level in the outer parts quickly falls to the noise level. For a laser power of 8μW, signal levels of at least 1 pA allow to clearly image the focus pattern, as depicted in Figure 2b (the corresponding topography map is depicted in Supporting Information S1: Figure S1). However, the quantum dots around the center have now been obviously damaged by high current levels, as better illustrated by the crosscut in Figure 2c (through *y* = 0 in the map in (b)). Outside the center region, the experimental data correspond very well to the calculated focus profile, plotted by the dotted red line in Figure 2c. For direct comparison, the calculated values were scaled by the normalizing power exponent of 0.64. We note that in basically all scans, dark (i.e., low current) spots or stripes are observed. We assume that this is related to quantum dot pickup and release by the probe tip, while no tip damage takes places. With regard to spatial resolution, as the width of the measured Airy features corresponds very closely to the calculation, we can quickly conclude that the experimental resolution is significantly below 200 nm. This value can be further reduced by comparing the measured focus pattern with calculated profiles including limited spatial resolution, which brings us to an upper limit for the spatial resolution at zero external bias of around 100 nm. On the other hand, when considering the absorption cross‐section of the quantum dots, the light excitation conditions, and the measured photocurrents, we can estimate a lower resolution limit of around 30 nm for zero external bias (see Supporting Information S1: Section 3).

**FIGURE 2:**
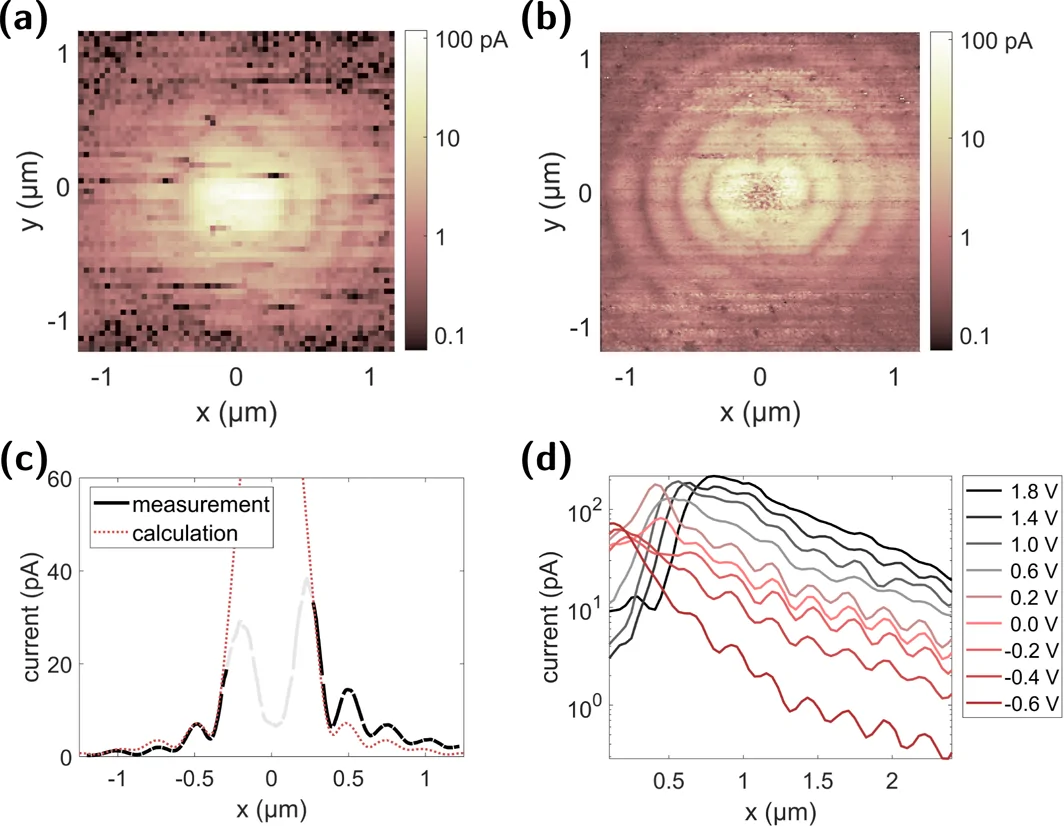
Tip scan pcAFM mapping of the focus of the 1.45 N.A. objective (wavelength 633 nm) on a quantum dot monolayer. Zero external bias was applied and the light intensity before the objective was (a) 0.4μW and (b) 8μW. (c) Current profile from (b) along y=0 (black/gray), compared to the numerically calculated intensity profile (red). The calculated values are scaled by a power exponent of 0.64 (and a constant), enabling the comparison of the calculated intensity values with the measured current data. (d) Extracted current profiles for x>0 from pcAFM maps for an incident laser power of 2μW and bias voltages of −0.6 to 1.8 V (for a built‐in voltage of 0.8±0.1 V).

Importantly, the latter consideration reveals that photocurrents as measured, that is, tens of pA, cannot be solely generated by a single quantum dot, but are provided from within an area of the quantum dot monolayer with a radius of at least 30 nm around the pcAFM tip contact. The achievable spatial resolution is thus bias‐dependent, which lets us to expect that for nearly compensated built‐in‐bias conditions, a resolution corresponding to the tip/sample contact area, that is, a few nm across can be achieved. While verifying the actual resolution requires light field structures in the nanometer range (which is beyond the scope of this work), we do investigate the role of nonzero external bias values on the photocurrent maps of the focus. The photocurrent cross‐sections derived from bias‐dependent (−0.6 to 1.8 V) photocurrent mapping of the focus are summarized in Figure 2d. Here, only values on the right side of the symmetry position (at *x* = 0) are shown on a logarithmic scale. Negative external bias values cancel the built‐in voltage and the photocurrent values outside the central lobe decrease. Conversely, the current within the central lobe increases, indicative of reduced damage to the quantum dots there. This is confirmed when turning the external bias to positive values, when the current in the central lobe is minimized. On the other hand, the current levels in the outer focus region increase and the Airy pattern contrast is lost. The latter effect is due to the increasing contribution of the neighboring quantum dots to the current probed at a specific position, ultimately smearing out the focus pattern contrast.

The irradiance dependence of the photocurrent is further demonstrated in Supporting Information S1: Figure S4 with stationary time‐resolved photocurrent measurements and photocurrent maps. These show that the damage occurring for elevated irradiance is not reversible (on the scale of tens of minutes). As we estimate that the local temperature increase in the sample for excitations up to 8 μW is below 10 K, we conclude that the quantum dot damage is not thermal. At this point, we cannot identify the process underlying the degradation but hypothesize that electrochemical reactions play a role. Further, the moderate temperature increase lets us exclude significant thermoelectric effects in our samples, which is supported from the former observation that the open‐circuit voltage on such systems is independent on the illumination power (see Figure 2b in ref. [3]).

### 3.3 Photocurrent Mapping of Plasmonic Lattice Field

We now turn to the light field distribution around quantum‐dot‐covered regularly arranged plasmonic gold nanocuboids. We limit our analysis to the area in between the particles, which here serve only as coherent scatter centers. The absorbance spectra of the nanocuboid array are plotted in Figure 3a for the polarization directions as indicated. The laser wavelength of 633 nm coincides with the plasmon resonance along the short (horizontal) cuboid axis. The array is imaged in sample scan mode, that is, the tip is fixed in the center of the focus of the 1.45 N.A. objective and the sample is scanned. The laser power is 0.3 μW, where no quantum dot bleaching is expected.

**FIGURE 3:**
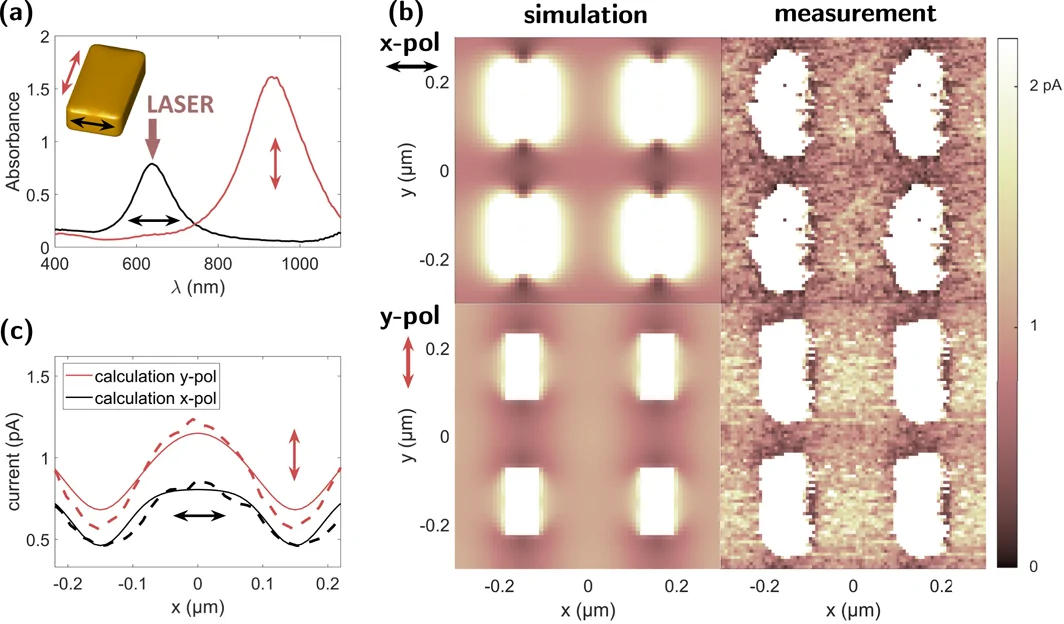
Sample scan pcAFM mapping of the gold nanocuboid array covered with a quantum dot monolayer. (a) Polarization‐dependent absorbance of the nanocuboid array, the inset shows the sketch of a single particle and the polarization directions. (b) Photocurrent maps at zero external bias for *x*‐ and *y*‐polarization, both measured and simulated. Note that the simulations do not include the quantum dot layer. The measured images show the average of 10 different unit cell area maps. The resulting averaged unit cell area is duplicated in a 2×2 matrix for clarity. The color map is optimized for the area on the bare substrate, and the particle area is thus saturated. The light intensity values in the numerically simulated maps were normalized by the power exponent of 0.64 for comparison with the photocurrent values. (c) Cross‐cuts from the maps in (b), the polarization directions are indicated by the arrows. The full lines are the theoretical profiles and the dashed lines are the measured profiles at *y* = 0.

The light field distribution between the cuboids is set by the coherent dipole scattering of the particles  [14, 15]. As no lattice resonance occurs for the given array parameters and laser wavelength  [14], we expect rather modest intensity variations that challenge our measurement scheme. In addition, the quantum dot layer exhibits a higher quantum dot agglomeration density in structured areas than on an unstructured surface, as shown in Supporting Information S1: Figure S1. The low current contrast together with agglomeration‐induced current variations within the array impose the need for averaging (over 10 individual maps) of small sample areas (single unit cells). Although a unit cell evidently contains the full information about the array, for the clarity of presentation, we duplicate the same map in a 2×2 matrix in Figure 3b for both polarization directions. The color scale in the maps is optimized for the area on the bare substrate, resulting in a saturated representation for the current on top of the gold particles (which are not considered in this work). Photocurrent maps with a globally adapted color scale are shown in Supporting Information S1: Figure S5. Next to the experimental maps in Figure 3b, we plot numerically simulated power‐law‐normalized intensity maps. Although subtle, the current variations in the flat sample regions agree well for experiment and simulation. For regions where no particle has to be crossed by the scanning tip, we find excellent agreement, as further illustrated by the current cross‐cuts in Figure 3c, extracted along *y* = 0 in the maps. There is a slightly larger deviation together with some stripe‐like features in areas where the tip is scanning over the 40‐nm high particles, related to feedback instabilities and contact condition variations. We conclude that the light distribution in the rather complex setting of the nanocuboid array is correctly mapped in the area in between the particles by pcAFM. We add that the quantum dot layer was not included in the simulation due to computational constraints, which is likely responsible for the differences between the experiment and simulation for the area close to the nanocuboids for x‐polarization.

## 4 Conclusion

In summary, perovskite‐capped PbS quantum dots provide photocurrents with a power‐law‐dependence on the illuminating light irradiance. The quantum dots also allow for a stable electric contact to metallized AFM tips, enabling spatially resolved photocurrent and thus isotropic light field mapping. The spatial resolution in the range of 30–100 nm for zero external bias is expected to extend both to lower and higher values, depending on the applied bias. The photocurrent maps of a laser focus and a plasmonic nanoparticle array agree very well to the simulated patterns. Photocurrent AFM in combination with quantum dot monolayers seems thus a promising detection scheme. Improvements can be expected, on one hand, from tailored quantum dots for specific charge generation and transport properties. On the other hand, better understanding the processes determining the spatial resolution could lead to improvements in this respect.

## Author Contributions

**Florian Küstner:** writing – review and editing, validation, formal analysis, experimental work, data analysis and modelling. **Andreas Hohenau:** writing – review and editing, validation, support on the experimental setup and data analysis. **Harald Ditlbacher:** writing – review and editing, validation, support on the experimental setup and data analysis. **Joachim R. Krenn:** conceptualization, funding acquisition, supervision, resources, project administration, writing – review and editing, validation.

## Funding

The authors acknowledge financial support by the University of Graz.

## Conflicts of Interest

The authors declare no conflicts of interest.

## Supporting information


Supporting Information S1

## Data Availability

All analyzed and discussed data are shown in the article or the Supporting Information S1.
