# Supplementary material for: Quantum‐Dot‐Mediated Light Field Imaging by Photocurrent Scanning Force Microscopy
Source: Nanophotonics. 2026 Mar 24;15(6):e70057. doi: 10.1002/nap2.70057 (PMC13642547; doi:10.1002/nap2.70057)
Supplement: Supplementary file 1 [file NAP2-15-e70057-s001.pdf]

Supplementary Material
Quantum-dot-mediated light field imaging by photocurrent scanning force microscopy

FLORIAN KÜSTNER,[1] ANDREAS HOHENAU,[1] HARALD DITLBACHER,[1] AND JOACHIM R. KRENN[1,*]

[1] *Institute of Physics, University of Graz, 8010 Graz, Austria*
[*] *joachim.krenn@uni-graz.at*

1. Sample topography

Figure S1(a) shows the topography map corresponding to the current map in Figure 2(b) (main text). The area under consideration is uniformly covered with quantum dots, with a few protruding features, presumably due to quantum dot aggregation. Figure S1(b) shows a typical topography map of the nanocuboid array used to measure the averaged current maps in Figure 3(b) (main text). In between the particles, some quantum dot inhomogeneities are visible that give rise to inhomogeneities in the current maps. These features can be suppressed by averaging over several unit cells of the cuboid array, as described in the main text.

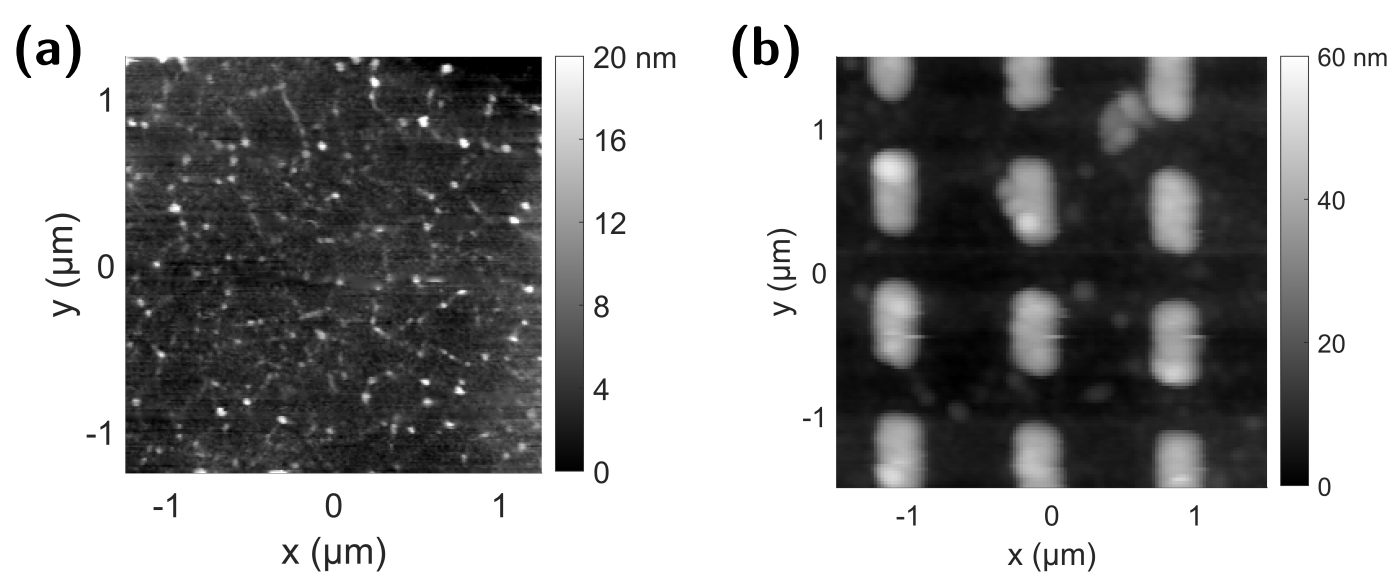

Fig. S1. (a) Topography map acquired together with the current map in Figure 2(b) (after line-wise mean height value subtraction to improve the visibility of the quantum dot agglomerates). (b) Typical topography map of the nanocuboid array used to measure the current maps in Figure 3(b) (without any image processing).

2. Built-in voltage

Considering the voltage dependence of the measured photocurrents in Figure 2(d) in the main text for x-values between 1.0 and 1.4 μm, we reconstruct an I/V relation that yields a built-in voltage of 0.8 ± 0.1 V, as plotted in Figure S2. This is in good agreement with the value of 0.7 ± 0.1 V found previously by bias sweeps on a similar sample with a similar probe tip [1]. The linearity of the current-voltage characteristic confirms that there is no sample deterioration in the considered focus area and bias range.

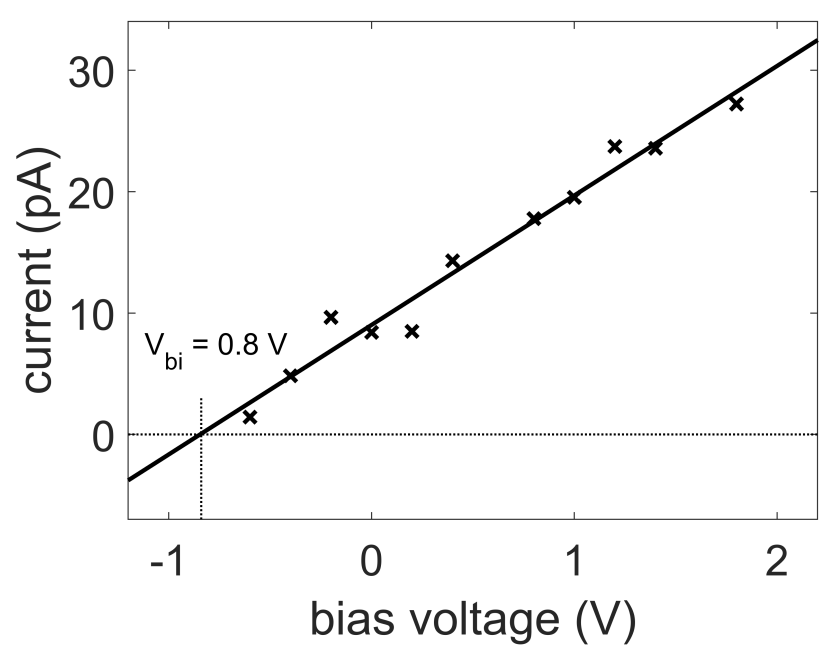

Fig. S2. I/V relation extracted from tip scans through a focused laser beam (symbols, shown together with the regression line). The current values are averages of the measured photocurrents plotted in Figure 2(d) in the main text for x-values between 1.0 and 1.4 μm.

3. Estimating the imaging resolution

Our finding that the spatial resolution is voltage-dependent (see Figure 2(d) in the main text) indicates that charge transfer between quantum dots and thus current-averaging over an area around the tip-contact is the resolution-limiting effect. In Figure S3(a), we compare a section of the measured focus profile in Figure 2(c) (main text) with calculated power-law-normalized profiles that are convoluted with spatial resolution values between 15 and 200 nm, as listed in the legend. We find that for an assumed resolution value of 100 nm the calculated pattern deviates clearly from the measured data, while values of 15 and 50 nm agree both well with the experimental data for the investigated system, allowing no further discrimination. We thus conservatively assume an upper spatial resolution limit of 100 nm under zero external bias.

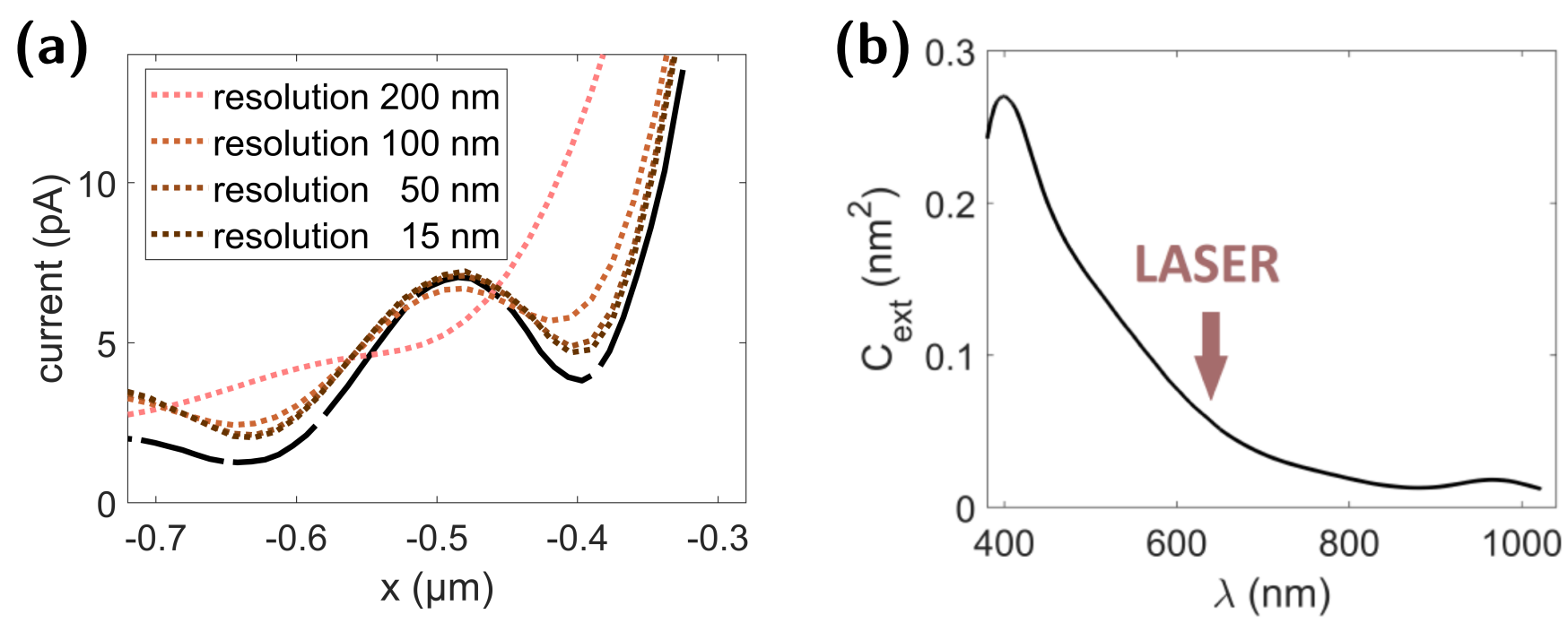

Fig. S3. (a) Section of the measured focus profile from Figure 2(c) (black), compared to calculated profiles assuming spatial resolution values as given in the legend. (b) Optical extinction cross section C_{ext} of a single PbS/$MAPbI_3$ quantum dot, as derived from the optical extinction of a PbS/$MAPbI_3$ solution. The arrow indicates the laser wavelength of 633 nm.

To estimate the lower limit of the resolution, we start with the the extinction cross section of a single PbS/$MAPbI_3$ quantum dot (Figure S3(b)), as derived from the extinction spectrum of a solution of 20 mg/L quantum dots in propylene carbonate, measured in a 4 mm wide glass cuvette. The quantum dots with a geometric cross section of 8.0 nm^2 show an extinction cross section of 0.06 nm^2 at an excitation wavelength of 633 nm. As the quantum dot extinction is dominated by absorption [2], we assume the same value for the absorption cross section. For an illumination irradiance of 300 W/cm^2, as present in the center of the focus in Figure 2(a) (main text), this corresponds to an excitation rate per quantum dot of about 10^6 electrons per second. This equals a maximum current of 0.2 pA, assuming that all absorbed photons give rise to a photocarrier. As evident from Figure 2 in the main text, the measured current for such illumination conditions is significantly above this value for a single quantum dot. This again evidences that photoexcited electrons from neighboring quantum dots are laterally transported within the monolayer to contribute to the measured current. As a rough estimate, the absorbed photons of about 400 quantum dots (corresponding to a sample area with a radius of about 30 nm) need to contribute to generate the measured currents of up to 80 pA. Taking the previous considerations into account, we can thus specify the lower limit for the resolution limit under zero external bias as 30 nm.

4. Quantum dot damage

Figure S4 (a,b) depicts time series of the photocurrent measured at a single spot of a quantum-dot-covered ITO surface. All measurements were done with an excitation wavelength of 633 nm and focusing through a 10×, numerical aperture 0.3 objective. In the first ten seconds the laser was blocked, giving zero current. For moderate laser excitation power (2 and 9 μW), the observed current is rather stable over time, but fluctuations increase with excitation power. For a laser excitation at 900 μW, however, initial strong fluctuations are followed by the disappearance of the photocurrent. Subsequent measurements under moderate conditions also show no current, indicating that the quantum dots were permanently damaged. In Figure S4 (c,d) sample scan pcAFM images of a previously damaged spot are shown. The map in (c) was acquired immediately after damaging, while the map in (d) was probed 20 minutes later. This shows that the damaged quantum dots do not recover (on this timescale).

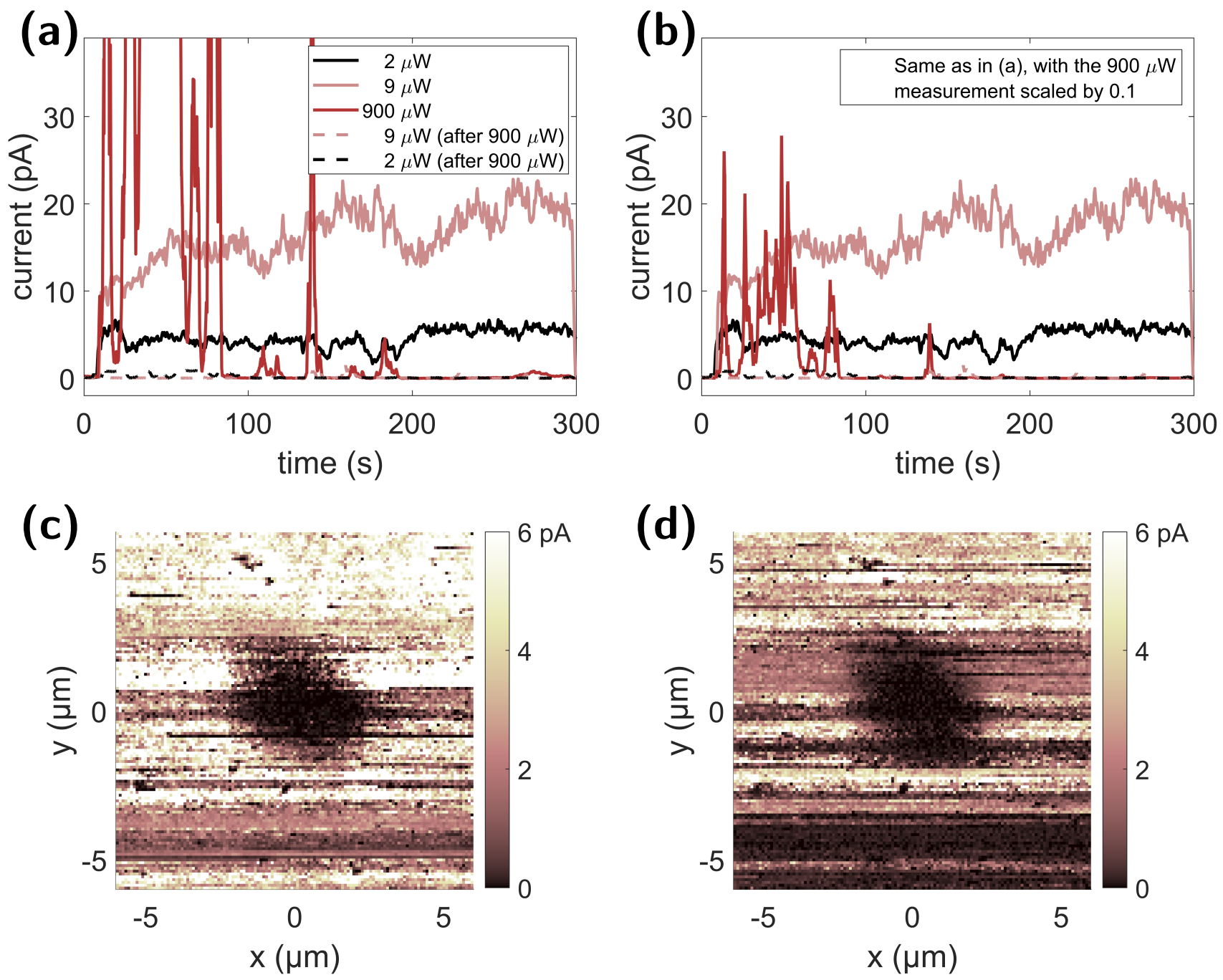

Fig. S4. (a) Photocurrent time series measured in the focus of a 10×, numerical aperture 0.3 objective for laser powers of 2 (black), 9 (brown) and 900 μW (red). The dashed black and dashed brown lines depict measurements for 2 and 9 μW, respectively, done after the 900 μW measurement. (b) Same as (a), with the 900 μW measurement scaled by a factor of 0.1. (c) Photocurrent map, acquired immediately after the area was exposed to 900 μW power. (d) Same as (c), but acquired 20 minutes later. (c) and (d) were measured in sample scan mode for a laser power of 9 μW.

5. Nanocuboid array photocurrent and topography maps

The color scale of the photocurrent maps in Figure 3(b) is optimized for the contrast in the area between the gold nanocuboids (which are not studied here). For the sake of completeness, we include here the same maps with globally adapted color scales. Accordingly, Figure S5 (a,b) show the experimental photocurrent maps in Figure 3(b) in the main text for x- and y-polarization, respectively. In panels (c) and (d) the corresponding averaged height maps are shown. For comparability with the representation in the main text and to emphasize the regular experimental arrangement of the nanocuboids, all averaged unit cells are duplicated on a 2×2 grid.

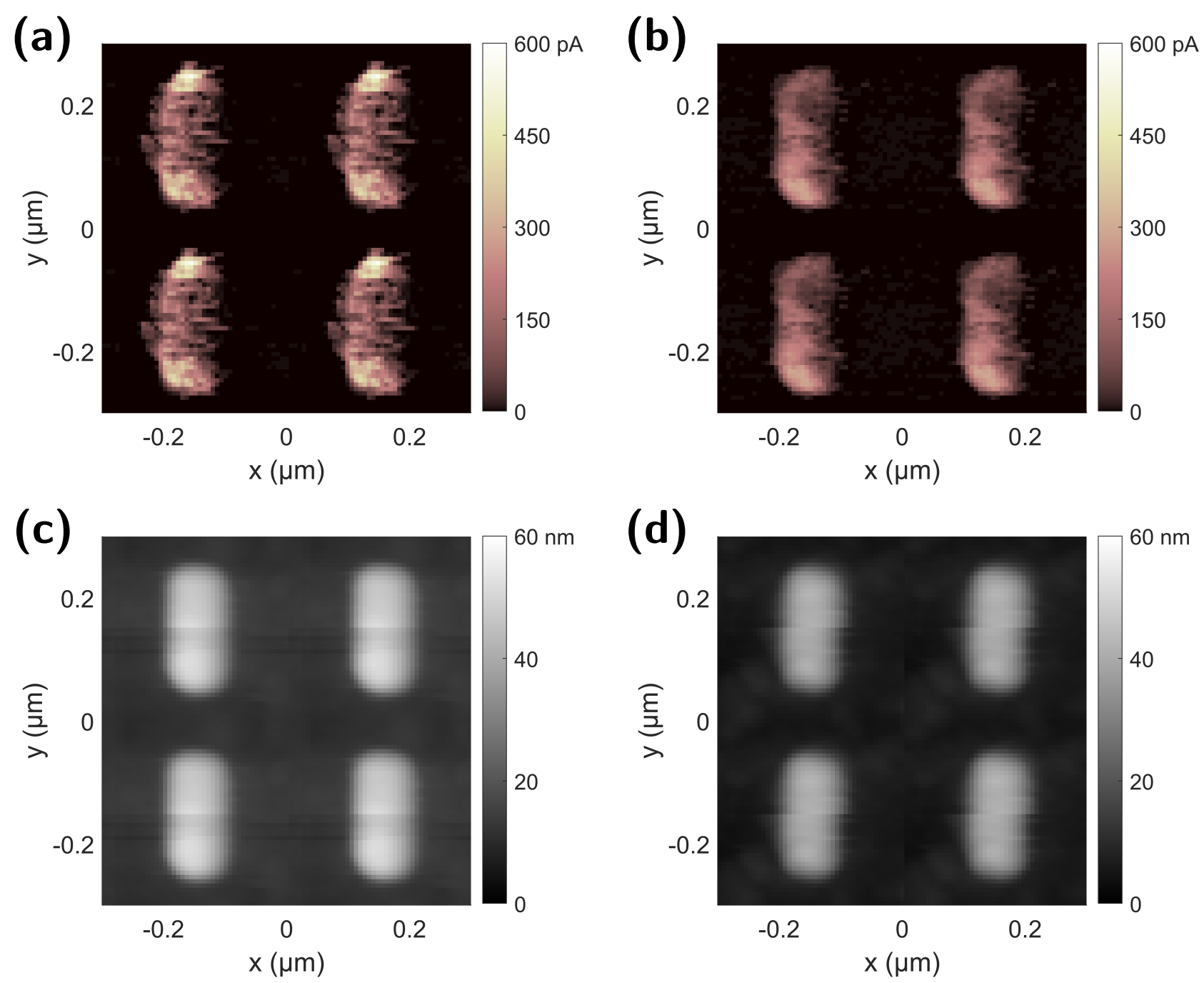

Fig. S5. (a) and (b) show the experimental photocurrent maps for x- and y-polarization, respectively, from Figure 3(b) (main text) with a globally adapted color scale. (c) and (d) depict the corresponding averaged topography maps.

References

1. F. Küstner, H. Ditlbacher, A. Hohenau, *et al.*, "Quantitative photocurrent scanning probe microscopy on PbS quantum dot monolayers," Nanoscale **16**, 16664–16670 (2024).
2. K. Xu, W. Zhou, and Z. Ning, "Integrated structure and device engineering for high performance and scalable quantum dot infrared photodetectors," Small **16**, 2003397 (2020).
